# Novel Molecular-Dynamics-Based Protocols for Phase Space Sampling in Complex Systems

**DOI:** 10.3389/fchem.2018.00495

**Published:** 2018-10-17

**Authors:** Sebastian Mai, Hugo Gattuso, Antonio Monari, Leticia González

**Affiliations:** ^1^Faculty of Chemistry, Institute of Theoretical Chemistry, University of Vienna, Vienna, Austria; ^2^Université de Lorraine and CNRS, LPTC UMR 7019, Nancy, France

**Keywords:** molecular dynamics, phase space sampling, simulation of absorption spectra, initial condition generation, quantum mechanics/molecular mechanics

## Abstract

The adequate exploration of the phase space of a chromophore is a fundamental necessity for the simulation of their optical and photophysical properties, taking into account the effects of vibrational motion and, most importantly, the coupling with a (non-homogeneous) molecular environment. A representative set of conformational snapshots around the Franck-Condon region is also required to perform non-adiabatic molecular dynamics, for instance in the framework of surface hopping. Indeed, in the latter case one needs to prepare a set of initial conditions providing a meaningful and complete statistical base for the subsequent trajectory propagation. In this contribution, we propose two new protocols for molecular dynamics-based phase space sampling, called “local temperature adjustment” and “individual QM/MM-based relaxation.” These protocols are intended for situations in which the popular Wigner distribution sampling procedure is not applicable—as it is the case when anharmonic or nonlinear vibrations are present—and where regular molecular dynamics sampling might suffer from an inaccurate distribution of internal energy or from inaccurate force fields. The new protocols are applied to the case of phase space sampling of [Re(CO)_3_(Im)(Phen)]^+^ (im, imidazole; phen, phenanthroline) in aqueous solution, showing the advantages and limitations of regular Wigner and molecular dynamics sampling as well as the strengths of the new protocols.

## 1. Introduction

Photochemical and photophysical phenomena have constantly gained importance in the last decades in many areas of research and technology. For example, in biological chemistry such processes play a decisive role in the interaction of sunlight with organisms—ranging from photosynthesis (Nelson and Ben-Shem, 2004), vision (Rando, 2001), or photomorphogenesis (Xu et al., 2015) to radiation-induced damage of biological tissue (Sinha and Häder, 2002; Holick, 2016). Additionally, bioluminescence (Navizet et al., 2011) and a large array of biomolecular imaging techniques (Lichtman and Conchello, 2005; Scott et al., 2011) are fundamentally based on photophysical processes. Hence, there is a strong drive to understand these processes with atomistic detail.

From the side of theoretical and computational chemistry, among the most relevant approaches to describe photochemical and photophysical phenomena are the simulation of UV-vis absorption spectra (Crespo-Otero and Barbatti, 2012) and performing molecular dynamics (MD) simulations, especially nonadiabatic *ab initio* molecular dynamics (AIMD) that predict the dynamics in the excited states occurring after photoexcitation (Lasorne et al., 2011). The information that can be gained through these simulations include the number and character of electronically excited states, their lifetimes, and the quantum efficiency connected to various photochemical reactions (Tully, 1990; Persico and Granucci, 2014). Furthermore, the dynamics simulations allow following the coupled nuclear motion and evolution of the electronic wave functions. In that sense, such simulations provide an approach to study and model photophysical properties that are complementary to experiments such as time-resolved pump-probe spectroscopy (Petit and Subotnik, 2014; Arbelo-González et al., 2016; Ruckenbauer et al., 2016; Crespo-Otero and Barbatti, 2018).

Naturally, a fundamental ingredient to nonadiabatic AIMD simulations is the description of nuclear motion. The latter is also important in the simulation of absorption spectra, allowing to obtain realistic band shapes and possibly band structures (Crespo-Otero and Barbatti, 2012; Nogueira and González, 2018). For large systems, such as the ones targeted here, nuclear motion is typically described classically, as the effort for a quantum-mechanical treatment scales exponentially with the number of degrees of freedom (DOFs) (Meyer et al., 2009). This classical nuclear motion typically follows forces from either a parameterized force field (FF) or an *ab initio* calculation. The latter case is called mixed quantum-classical dynamics (Tully, 1998). For excited-state nonadiabatic AIMD simulations in polyatomic molecules, most often the surface hopping (SH) method (Tully, 1990; Crespo-Otero and Barbatti, 2018) is applied.

One serious consequence of the classical approximation is that a delocalized nuclear wave function—with nuclear density spread over a region of coordinate space—is replaced by point-like, completely localized trajectories. Naturally, this means that a single trajectory cannot represent the full dynamics of a molecule. Instead, it is more appropriate to replace the nuclear density by an ensemble consisting of a large number of point-like trajectories. A statistical analysis of this ensemble then allows retrieving information on the behavior of the actual wave packet.

A logical consequence of such a statistical approach is that it is necessary to obtain a large set of point-like initial conditions—geometries and momenta—from which the ensemble of trajectories can be started. This set of initial conditions should naturally provide a good representation of the distribution of nuclear geometries and momenta corresponding to the initial state of the system (Barbatti and Sen, 2016). Even if one is only interested in simulating absorption spectra, employing a distribution of geometries can be beneficial to obtain the approximate contours of the absorption bands, e.g., its asymmetry or shift of absorption maximum vs. vertical excitation energy (Bergsma et al., 1984; Crespo-Otero and Barbatti, 2012; Kossoski and Barbatti, 2018; Zobel et al., 2018). This is especially important in biological systems (Etienne et al., 2013; Nogueira and González, 2018). In order to obtain the mentioned set of distributed geometries and momenta, two main techniques to *sample the nuclear density distribution* have been adopted by the scientific community (Barbatti and Sen, 2016; Zobel et al., 2018): (i) Sampling from the Wigner distribution (we will call this “quantum sampling” here) of an approximate quantum-mechanical nuclear wave function (Dahl and Springborg, 1988; Schinke, 1995), and (ii) sampling via (adiabatic) MD simulations (“classical sampling”) (Bergsma et al., 1984). Both approaches have advantages and limitations. Quantum sampling produces a better representation of the true nuclear density, but is limited to rather small systems. Classical sampling, on the contrary, does not yield a good description of zero-point energy, but correctly includes anharmonicities and a distribution over many local minima with similar energies (Etienne et al., 2013; Nogueira and González, 2018), both of which are difficult to treat with quantum sampling.

In this contribution, we report two protocols designed to produce a meaningful representation of initial conditions in terms of both geometries and velocities for general flexible chromophores embedded in complex environments. Our protocols are based on classical sampling, but attempt to correct for some of the deficiencies of that sampling method. They are discussed in detail together with a critical review of the quantum and classical sampling techniques and their advantages and limitations. The protocols are applied to the transition metal complex *fac*-[Re(CO)_3_(Im)(Phen)]^+^ (Im, imidazole; Phen, phenanthroline) in a box of water molecules. This complex has been used in the last decades as a chromophore in the study of long-range electron transport in proteins (Winkler and Gray, 1992; Wuttke et al., 1992; Warren et al., 2012) and also has been investigated without a protein in solution (Vlček, 2009; El Nahhas et al., 2011). In this regard, one of our goals is to generate a realistic ensemble of initial conditions for [Re(CO)_3_(Im)(Phen)]^+^, intended for use in absorption spectrum simulations, as well as for nonadiabatic AIMD simulations of the intersystem crossing dynamics of this complex. Our sampling strategy will be validated by the proper reproduction of the main spectroscopic properties and the distribution of the most important DOFs.

## 2. Computational details

The computations involved two main steps. In the first step, the ground state phase space distribution of [Re(CO)_3_(Im)(Phen)]^+^ was sampled with different methods—quantum sampling (Wigner distributions) and classical sampling (using MD simulations)—as will be discussed in detail in section 3. In the second step, we compute UV absorption spectra based on the different sampled ensembles using time-dependent density functional theory (TD-DFT).

### 2.1. Sampling

Sampling using Wigner distributions has been performed based on frequency calculations for all ground state minima of [Re(CO)_3_(Im)(Phen)]^+^ (see section 3), using B3LYP/TZP (Becke, 1993; van Lenthe and Baerends, 2003) and COSMO (Klamt and Schüürmann, 1993; Pye and Ziegler, 1999) to mimic the environment of an aqueous solution. For each ground state minimum (see section 1 in the Supplementary Material for coordinates), quantum sampling was used to generate several hundred geometries, such that the ratios between the minima reflects their energy differences (see below). The final ensemble from quantum sampling was then obtained as the union of the sampled geometries for all minima. We note that the quantum sampling was carried out at 300 K, by stochastically choosing a vibrational level from the Boltzmann distribution at that temperature and then sampling from the Wigner distribution corresponding to the chosen level.

Classical sampling was based on MD simulations carried out with AMBER16 (Case et al., 2017). The used FF parameters for the metal complex were the same as in our previous publication (Mai et al., 2017). Briefly, the ligand bond, angle, and dihedral parameters were taken from the generalized Amber force field (Wang et al., 2004; Pérez et al., 2007), while the missing parameters involving the Re atom were parameterized ad hoc and refined after carrying out a few-ps QM/MM (hybrid quantum mechanics/molecular mechanics) trajectory. For Re, the Van-der-Waals radius was set to 1.47 Å, and the electrostatic non-bonded term to 0.241, as given by Oliveira et al. (2013). Point charges for [Re(CO)_3_(Im)(Phen)]^+^ were computed using a RESP fit (Bayly et al., 1993) to electron density obtained at the B3LYP/6-31G^*^ (LAN2DZ for Re; Hay and Wadt, 1985) level of theory, using the Gaussian09 software (Frisch et al., 2009).

The full system for the MD simulations consisted of [Re(CO)_3_(Im)(Phen)]^+^ surrounded by a 12 Å truncated octahedron box of 1,054 H_2_O molecules represented by the TIP3P model (Jorgensen et al., 1983) and a chloride ion. After minimization for 4000+4000 steps (steepest descent and conjugate gradient, respectively) and thermalization to 300 K (NVT ensemble) for 20 ps, the system was equilibrated to 1 bar for 200 ps (NPT ensemble). The 500 geometry samples—making up the classical sampling ensemble—were taken every 20 ps from the production run of 10 ns (NPT ensemble). All simulations employed a time step of 0.5 fs. This relatively short time step was employed because we wanted to avoid constraining (e.g., using the SHAKE algorithm) the C–H and N–H bond vibrations of the complex.

### 2.2. Electronic structure calculations

The electronic structure calculations employed the B3LYP functional (Becke, 1993) with the ZORA relativistic Hamiltonian (van Lenthe et al., 1993), as implemented in the ADF2017 package (Baerends et al., 2017). This level of theory has been shown (Daniel, 2015, 2016; Fumanal and Daniel, 2016a) to deliver a satisfactory description of the electronically excited states of Re(I) complexes similar to [Re(CO)_3_(Im)(Phen)]^+^. The basis set for the Re atom was ZORA-TZP (van Lenthe and Baerends, 2003), while all other atoms were treated with the ZORA-DZP basis set or the ZORA-DZ basis set (H atoms and all atoms of im except for the coordinating N); in the following, this basis set is denoted as “DZ(P).” No electrons were frozen and standard SCF settings were used. Interactions with the surrounding water were included through electrostatic embedding (Mai et al., 2017) for geometries obtained through classical sampling, and COSMO (linear-response non-equilibrium solvation Tomasi et al., 2005) for geometries from quantum sampling.

To achieve a good balance between computational efficiency and accuracy, we made use of the “locally dense grids” feature of ADF (Franchini et al., 2013), where different atoms can be assigned different numerical integration qualities. For the Becke numerical integration grid (Franchini et al., 2013), heavy atoms were treated with good quality, whereas H atoms used normal quality. For the ZlmFit Coulomb fit method (Franchini et al., 2014), the metal atom, the first coordination sphere, and the oxygen atoms used good quality, other heavy atoms used normal quality, and hydrogens used basic quality. Finally, for the RI Hartree-Fock scheme (Krykunov et al., 2009)—which is the main bottleneck in ADF calculations with hybrid functionals—basic quality was used for heavy atoms and “verybasic” quality for H atoms. As demonstrated in the Supplementary Material (section 2, Figure S1, Table S1), these settings lead to a speed-up of about 100%, while producing errors in energies of 0.02 eV and relative errors in gradients of about 1%. Note that the frequency calculations needed for quantum sampling were performed with more accurate settings.

The vertical excitation calculation for the spectrum simulations employed the Tamm-Dancoff approximation (TDA) to compute 30 singlet states. Since it has been shown that spin-orbit couplings do not notably affect the spectral shape of [Re(CO)_3_(Im)(Phen)]^+^ (Mai et al., 2017), here we only report spin-free absorption spectra.

### 2.3. Absorption spectrum

The absorption spectra were computed from the excitation energies *E*_*gi*_ and oscillator strengths (_*f*_osc_)*gi*_ between ground state and excited state *i* obtained from all sampled geometries *g*. The total spectrum is given by a sum over Gaussians centered at *E*_*gi*_ and with height proportional to (_*f*_osc_)*gi*_:

(1) $$\sigma (E)=\sum_{g}^{n_{\text{geom}}}\sum_{i}^{n_{\text{state}}}{\left(f_{\text{osc}}\right)}_{gi}\cdot \exp\left(-4\ln(2)\frac{{\left(E-E_{gi}\right)}^{2}}{{\text{FWHM}}^{2}}\right).$$

A full-width at half-maximum (FWHM) of 0.15 eV was employed in order to smooth the spectra.

In order to estimate the influence of the random sampling on the shape of the absorption spectrum, we applied the bootstrapping procedure (Nangia et al., 2004). In this procedure, we randomly picked (with replacement) *n* geometries from the respective ensemble of *n* geometries to generate a “bootstrap” ensemble, from which a new spectrum can be computed according to Equation (1). This random picking was repeated 100 times, yielding 100 slightly different spectra. The sampling error was then estimated from the standard deviation of these spectra. This bootstrapping procedure is implemented in SHARC2.0 (Mai et al., 2018).

## 3. Phase space sampling of [Re(CO)_3_(Im)(Phen)]^+^

### 3.1. Conformers of [Re(CO)_3_(Im)(Phen)]^+^

**Figure 1:**
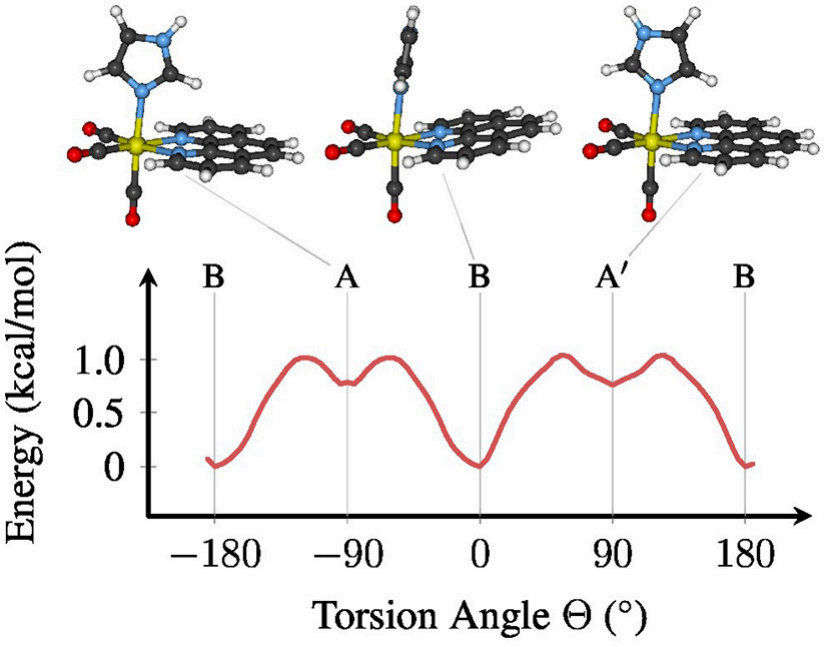
Ground state (*S*_0_) potential energy profile for the torsion around the Re–im bond (B3LYP/TZP, COSMO). The stable conformers *A*, *B*, and *A*′ are depicted above the plot. For a definition of the torsion angle Θ, see the Supplementary Material.

The metal complex *fac*-[Re(CO)_3_(Im)(Phen)]^+^ exhibits an almost free torsion around the single bond between the metal center and the im ligand (Baranovskii and Maltsev, 2014; Fumanal and Daniel, 2016a,b; Mai et al., 2017). Along this torsional mode, three stable minima in the electronic ground state can be optimized, as shown in Figure 1. Using the definition of the torsion angle Θ that we have employed in our previous publication (Mai et al., 2017) (see the Supplementary Material section 3 and Figure S2 for the definition), the most stable conformation of [Re(CO)_3_(Im)(Phen)]^+^ is conformer B, which is found in the crystal structure of [Re(CO)_3_(im)(phen)]_2_SO_4_·H_2_O (Connick et al., 1995). In this conformer, Θ is close to 0°, meaning that the im and phen ligands are oriented “parallel” to each other. The two other conformers assume a planar symmetry (*C*_*s*_), with im oriented perpendicular to phen. Conformer *A* is located at Θ equal to −90° and *A*′ at +90°.

As shown in Figure 1, the energy difference between the conformers, as well as the related barriers, are on the order of 1 kcal/mol. Hence, at room temperature the molecule can almost freely rotate around Θ, although the chiral conformer *B* will be the most abundant conformation, with about 75–80% abundance. This shows that all three conformers need to be properly included in any phase space sampling of [Re(CO)_3_(Im)(Phen)]^+^ and similar complexes.

### 3.2. Quantum sampling

We will first discuss the problems and limitations that arise when applying quantum sampling to a flexible molecule like [Re(CO)_3_(Im)(Phen)]^+^. By quantum sampling, here we specifically mean sampling from a Wigner distribution of the nuclear vibrational states of a quantum oscillator, with the potential around the minimum geometry described in the harmonic approximation (Dahl and Springborg, 1988; Schinke, 1995) in normal mode coordinates. The advantage of this procedure is that it provides a reasonably good representation of the vibrational density distribution (positions and momenta) as well as the zero-point energy for typical small to medium-sized molecules in the gas phase (Barbatti and Sen, 2016). Due to this accuracy, and the simplicity and efficiency for such systems, quantum sampling is widely used to produce initial conditions for nonadiabatic AIMD.

However, quantum sampling has a number of limitations, which restrict its applicability for larger systems. First, it is required to compute the Hessian matrix of the potential energy, which scales quadratically with the number of atoms in the system. Due to this scaling, it quickly becomes unfeasible to perform this computation.

Second, quantum sampling assumes that the PES can be expanded around a single minimum energy geometry (which needs to be optimized first). This assumption is typically well justified for many isolated molecular systems, whereas molecules with multiple local minima require that one samples randomly from a set of several Wigner distributions which are Boltzmann-weighted according to the energies of the respective minima (Kossoski and Barbatti, 2018). However, this approach is only feasible if the system possesses a small number of well-defined and well-separated minima. The situation becomes significantly more complex when an environment (e.g., solvent or biopolymer matrix) is explicitly included in the sampling. In this case, the system possesses an extremely large number of local minima with very similar energies, with each individual minimum—even the global one—contributing only very little to the accessible phase space. This is typically the case in systems that are treated with hybrid quantum mechanics/molecular mechanics (QM/MM) techniques.

Third, the quantum sampling approach is based on the assumption of a harmonic PES around each local minimum, which is also a bad approximation for large systems. Typically, this approximation is particularly severe for large-amplitude, low-frequency movements. Examples for such very anharmonic movements in molecular systems are out-of-plane deformation (bends) of extended π-conjugated systems or the torsional movement of monodentate ligands in transition metal complexes. These modes are present in [Re(CO)_3_(Im)(Phen)]^+^ due to the bending modes of the phen ligand and the aforementioned torsion of the im ligand. In a poly-molecular environment, additionally motions like rotation of solvent molecules or diffusion also tend to be very anharmonic.

Fourth, in order to solve the nuclear Schrödinger equation for a multi-dimensional harmonic oscillator, one typically employs linear normal mode coordinates to decouple the degrees of freedom. This leads to additional problems, because in (poly-)molecular systems many movements are highly non-linear, for example torsions, solvent rotation, or diffusion.

Here, we want to illustrate the influence of the two latter limitations—harmonic approximation and linear coordinates—on the description of the im torsion of [Re(CO)_3_(Im)(Phen)]^+^. To this end, we generated two independent quantum-sampled ensembles for [Re(CO)_3_(Im)(Phen)]^+^: one where the lowest harmonic vibrational mode—the im torsion at 7–10 cm^−1^—was neglected for the sampling, and one where it was included. In order to overcome the single-minimum limitation, both independent ensembles were created as the union of 200 geometries for conformer *A* (15%), 1,000 geometries for *B* (77%), and 100 geometries for *A*′ (8%), giving a total of 1,300 geometries. These values are based on the relative abundance of conformers *A*, *B*, and *A*′ reported previously (Mai et al., 2017): 14% in *A*, 77% in *B*, and 9% in *A*′. These two ensembles were compared to an ensemble consisting of the 500 snapshots taken from the classical MD simulation (as described in the section Computational Details).

**Figure 2:**
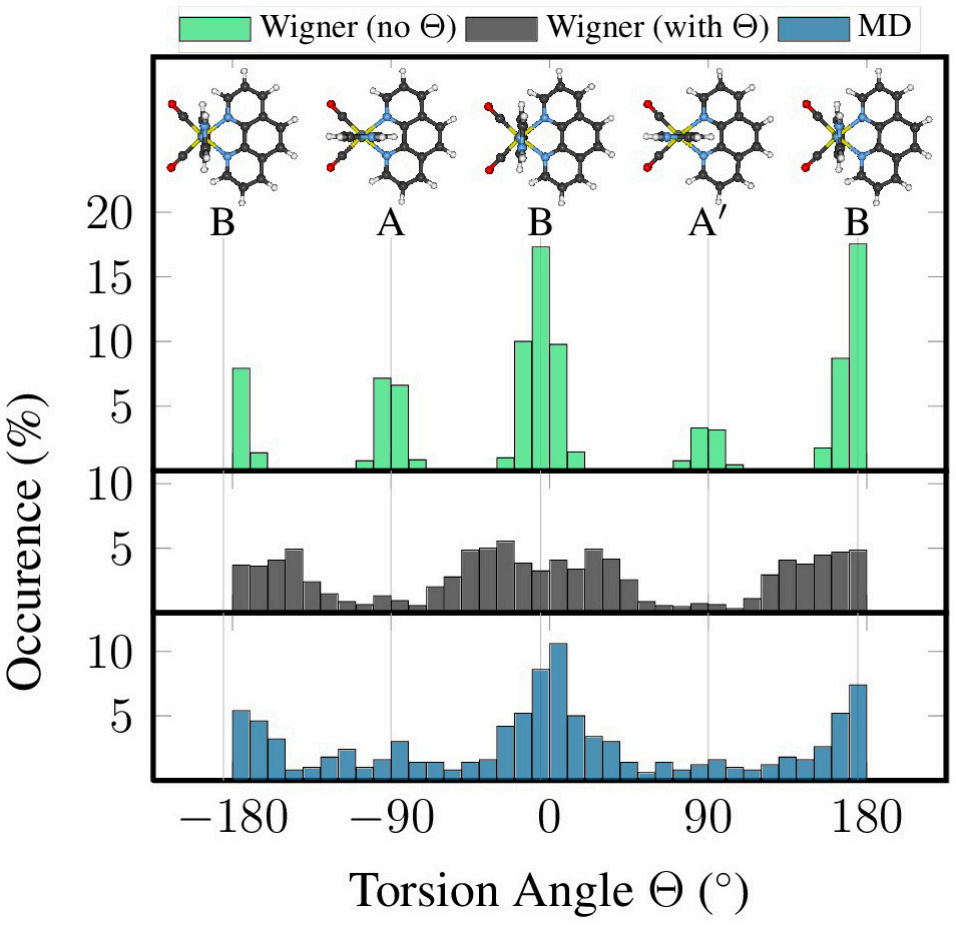
Histograms of the distribution of torsion angle Θ from quantum sampling—either neglecting the torsional normal mode (green) or including it (black)—and from classical sampling using a FF (blue).

Figure 2 presents histograms for the distribution of the torsion angle Θ of im, based on these three ensembles. It can be clearly seen that the “no Θ” sampling, where the torsion mode was not included, leads to very narrow distributions in the torsion angle, which is not in agreement with the flatness of the potential energy profile of this mode. On the contrary, the “with Θ” sampling produces a much wider distribution, which is actually in decent agreement with the distribution in the MD-based ensemble.

**Figure 3:**
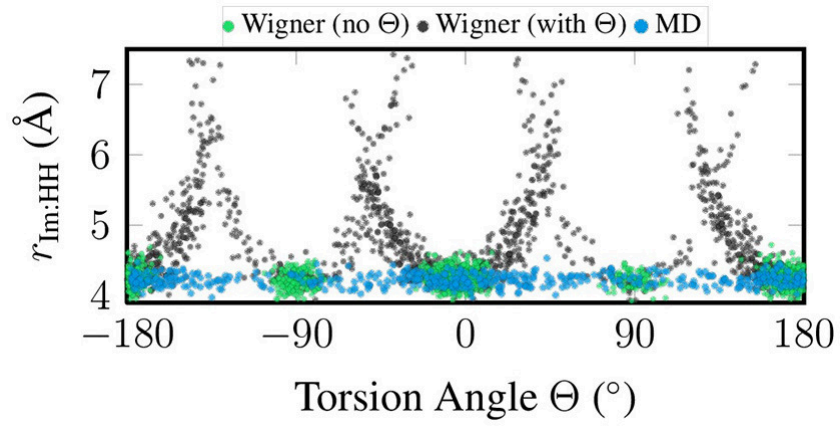
Scatter plot of torsion angle Θ against the distance between the two metal-facing H atoms of the im ligand, based on the geometries coming from quantum sampling—either neglecting the torsional normal mode (green) or including it (black)—and from classical sampling using a FF (blue).

Figure 3 presents a correlation plot between the sampled torsion angle Θ and the total width of the im ligand, which we measure as the distance between the two H atoms closest to the metal atom. In the figure, it can be seen that for the MD sampling, the width of im is independent of the torsion angle, which agrees fully with expectations. The same is true for the “no Θ” sampling, although there all geometries are clustered around the minimum torsion angles. On the contrary, in the “with Θ” sampling the im width depends strongly on the torsion angle, with angles far from the minima leading to strongly exaggerated ligand widths of up to 7 Å (compare to the equilibrium distance of 4.2 Å). The reason for this problem is that the Wigner sampling procedure is based on linear normal mode coordinates, which is a very bad approximation for the torsion. The actual normal mode vector of the normal mode displaces the hydrogen atoms on straight lines perpendicular to the molecular plane of im, such that the molecule is *sheared* instead of rotated. This shearing operation leads to the 1/cos(Θ−Θ_eq_) behavior seen in Figure 3.

At this point, we should emphasize that the problems evidenced in Figures 2,3 are not related to the sampling of the environment, but are intrinsic to the [Re(CO)_3_(Im)(Phen)]^+^ molecule, due to its free torsion mode. Hence, techniques which combines quantum sampling for the chromophore and classical sampling for the environment are not applicable to [Re(CO)_3_(Im)(Phen)]^+^. Such approaches are often called “hybrid initial condition methods,” and have been reported by Kästner et al. (2006) and Ruckenbauer et al. (2010). In the variant of Ruckenbauer et al. (2010), first classical sampling of the solvent environment around a frozen chromophore at the minimum geometry is performed, allowing to extract snapshots representative of different solvent conformations. Subsequently, a Wigner distribution of the chromophore is generated, and the frozen geometry in the MD snapshots is substituted by a Wigner-sampled geometry with corresponding momenta. While appealing for small molecules in homogeneous solutions, such an approach is not suitable for larger, flexible molecules—due to the limitations of linear harmonic oscillator quantum sampling—and neither for chromophores covalently bonded to their environment, as for example in DNA or proteins.

### 3.3. Classical sampling

For systems that can neither be sampled with Wigner techniques nor with the hybrid initial conditions methods, classical sampling via MD simulations is one of the few remaining alternatives. MD sampling in principle is well suited to explore in an appropriate way the phase space of a large (poly-) molecular system. First, all anharmonic motions, like torsions or diffusion, are fully included in the sampling, as long as the underlying description of the potential energy surface is realistic. Second, MD sampling automatically considers all local minima in the potential energy surface, and sufficient sampling can even spot minimum energy conformations which were missed by manual optimization.

Such classical sampling requires some method to provide the potential energy and its gradient for any relevant geometries. These energies and gradients can come from electronic structure calculations; in this case, one would speak about AIMD. However, electronic structure calculations are computationally expensive, making AIMD sampling of large systems over sufficiently long time scales unfeasible. Hence, often mixed QM/MM techniques are employed, whereby only the chromophore is described with electronic structure methods (QM) and the environment by classical FFs (MM). Unfortunately, even with this technique proper sampling of systems with several thousand atoms is not feasible, as very long simulations are required to sample all possible configurations of such large systems. Therefore, only FF-based classical sampling allows reaching the size and time scales required for the task at hand.

However, there are some disadvantages of FF-based MD sampling. First, it does not provide a good description of the partitioning of the internal energy among the DOFs. In particular, classical sampling ignores the fact that each mode *i* is required to carry at least the quantum-mechanical zero-point energy (ZPE) of $\frac{1}{2}\hbar \omega_{i}$ (Barbatti and Sen, 2016; Zobel et al., 2018)—or even more at elevated temperatures. Instead, each classical mode only contains energy (kinetic plus potential) that is proportional to the chosen temperature in the MD simulation, namely *k*_*B*_*T*. *k*_*B*_ times 300 K is about 26 meV, which is the ZPE of a normal mode with 417 cm^−1^. Hence, classical sampling on one hand produces a wrong distribution of energies—each mode receives the same amount independent of the frequency—and on the other hand a too low total internal energy for most molecules. Therefore, when applying classical sampling at 300 K for generating ensembles for spectrum and nonadiabatic AIMD simulations, one will find that the spectra are too narrow and the dynamics too slow (Klaffki et al., 2012; Barbatti and Sen, 2016; Zobel et al., 2018).

A second disadvantage of FF-based MD sampling is that one has to rely on the accuracy of the FF. Especially when using general FFs like GAFF (Wang et al., 2004; Pérez et al., 2007) or the CHARMM general FF (Vanommeslaeghe et al., 2009), it is possible that the MD simulations predicts different geometric parameters (bond lengths, angles, etc.) than a QM or QM/MM calculation would. This might introduce systematic errors in the spectrum and nonadiabatic AIMD simulations that employ geometries sampled with FF-based MD.

### 3.4. Novel protocols for classical sampling

In the following, we will describe the two novel approaches that we employ here to lessen the effect of the two above-mentioned limitations of FF-based MD sampling: inaccurate internal energy distribution and inaccurate description by the FF. The first limitation will be tackled by *local temperature adjustment*, whereas the second limitation is treated with *individual QM/MM-based relaxation*.

#### 3.4.1. Local temperature adjustment

In an MD simulation at a temperature of 300 K, all degrees of freedom of the system receive on average a thermal energy of 26 meV. For [Re(CO)_3_(Im)(Phen)]^+^, which has 38 atoms and therefore 108 internal degrees of freedom, this leads to a total thermal energy of about 2.8 eV. In contrast, the sum of the ZPE of all normal modes of [Re(CO)_3_(Im)(Phen)]^+^ is about 7.4 eV according to a frequency calculation of conformer *B* (using B3LYP/TZP). This value was verified by an anharmonic frequency calculation (Barone, 2005) using Gaussian 16 (Frisch et al., 2016) at the B3LYP/LANL2DZ + IEFPCM(water) level of theory, which yielded an anharmonic ZPE correction of only −0.08 eV. When neglecting the ZPE contributions of the H stretch modes, the ZPE is still 5.0 eV. Hence, the MD simulation significantly underestimates the total ZPE of the molecule. Moreover, the ZPE per mode should range from 0.1 to 225 meV, whereas it is a flat 26 meV in the MD simulation.

The problem of the inaccurate energy *distribution* is inherently tied to the classical description of the nuclei. Indeed, as was already stated by Stock and Müller (1999), “…there is no rigorous *and* useful solution to the ZPE problem ….” Hence, here we will not attempt to correct for the problem of energy distribution. Instead, we only aim at diminishing the effect of the too low internal energy that is given to [Re(CO)_3_(Im)(Phen)]^+^ by an MD simulation at 300 K. Equivalent to the ZPE of 7.4 eV of [Re(CO)_3_(Im)(Phen)]^+^, an appropriate temperature to carry out the MD simulations (Barbatti and Sen, 2016) would be around 800 K—or slightly below 600 K when neglecting the ZPE of the H stretch modes. However, it would be unrealistic to heat the complete system to 600 or 800 K, as that would also affect the translational and rotational DOFs of the environment. Hence, a compromise was sought by performing the all-atom MD simulations at 300 K and reheating only the metal complex locally to 600 K in a subsequent computation step. We term this approach *local temperature adjustment*. In the following, we describe the sequence of computational steps we employed.

The local heating procedure started at the 500 snapshots (geometries and velocities) that were obtained from the 10 ns NPT run described above. Then, for each individual snapshot, a follow-up MD simulation was performed. In these simulations, all water molecules and the chloride ion were frozen and the system was run for 20 ps with the thermostat set to 600 K. As applied pressure would not affect the frozen coordinates of the water, these simulations were carried out in the computationally simpler NVT ensemble, with periodic boundary conditions removed (after reimaging all coordinates to the primary cell). Since the solvent is frozen, effectively only the chromophore is heated to 600 K. Note that the chromophore is constrained to the frozen solvent cavity and should therefore not change its conformation too much. Subsequently, the velocities of the solvent atoms were reset to the values from the production snapshots, corresponding to 300 K.

Following the local heating procedure, we also applied a second MD simulation to the endpoints of the heating trajectory, in order to given the solvation shells of [Re(CO)_3_(Im)(Phen)]^+^ a brief period of time (100 fs) to adapt to the new conformation of the complex. The result of this re-equilibration are 500 snapshots of a water nano droplet (diameter 36 Å) surrounding a hot [Re(CO)_3_(Im)(Phen)]^+^ with an internal energy that is relatively close to its ZPE.

#### 3.4.2. Individual QM/MM-based relaxation

All steps described so far were carried out with MD with the classical FF described previously (Mai et al., 2017). In particular, the FF uses GAFF parameters for phen and im, and newly parametrized values for the bonds involving Re (Mai et al., 2017). While the bonds involving Re were parametrized from a ground state B3LYP calculation—and thus should give results in close agreement with a QM/MM trajectory—the parameters used for phen and im are not adapted specifically for these ligands. Consequently, GAFF might produce too long or short bond lengths or inaccurate angles, compared to a QM/MM calculation. This is disadvantageous, because it can lead to inaccurate excitation energies (Sánchez-Murcia et al., 2018) and large coherent motion after switching from the GAFF description (during sampling) to the QM/MM description (in the spectra and nonadiabatic AIMD simulations).

In order to reduce these FF influences, for each snapshot we performed a short QM/MM-MD trajectory in the ground state. In this way, fast DOFs like bond lengths and bond angles will relax toward the QM/MM equilibrium values. The slower DOFs will not adapt sufficiently in a short QM/MM-MD trajectory, but we assume that these DOFs were sampled adequately by the MD sampling, because the long-range parameters (point charges and Van-der-Waals parameters) of [Re(CO)_3_(Im)(Phen)]^+^ were specifically parametrized for this molecule.

We performed the QM/MM-MD simulations with the SHARC program, as it is also used for the subsequent spectra and nonadiabatic AIMD simulations. To this end, the coordinates and velocities from the last step were converted from AMBER restart format into SHARC initial condition format. Here, it was crucial to account for the fact that AMBER is using a leapfrog-like algorithm which stores in the restart file coordinates **R**(*t*) and velocities $\text{v}\left(t-\frac{\Delta t}{2}\right)$. This is problematic because SHARC is using the velocity Verlet algorithm and thus expects **R**(τ) and **v**(τ) at the same point in time. To compensate for this, during the format conversion we compute

(2) $$R(\tau )=R(t-\frac{\Delta t}{2})=R(t)-\frac{\Delta t}{2}v(t-\frac{\Delta t}{2}),$$

where Δ*t* was the time step employed in the AMBER simulations (0.5 fs). After these steps, we obtained a set of 500 initial conditions in SHARC format.

**Figure 4:**
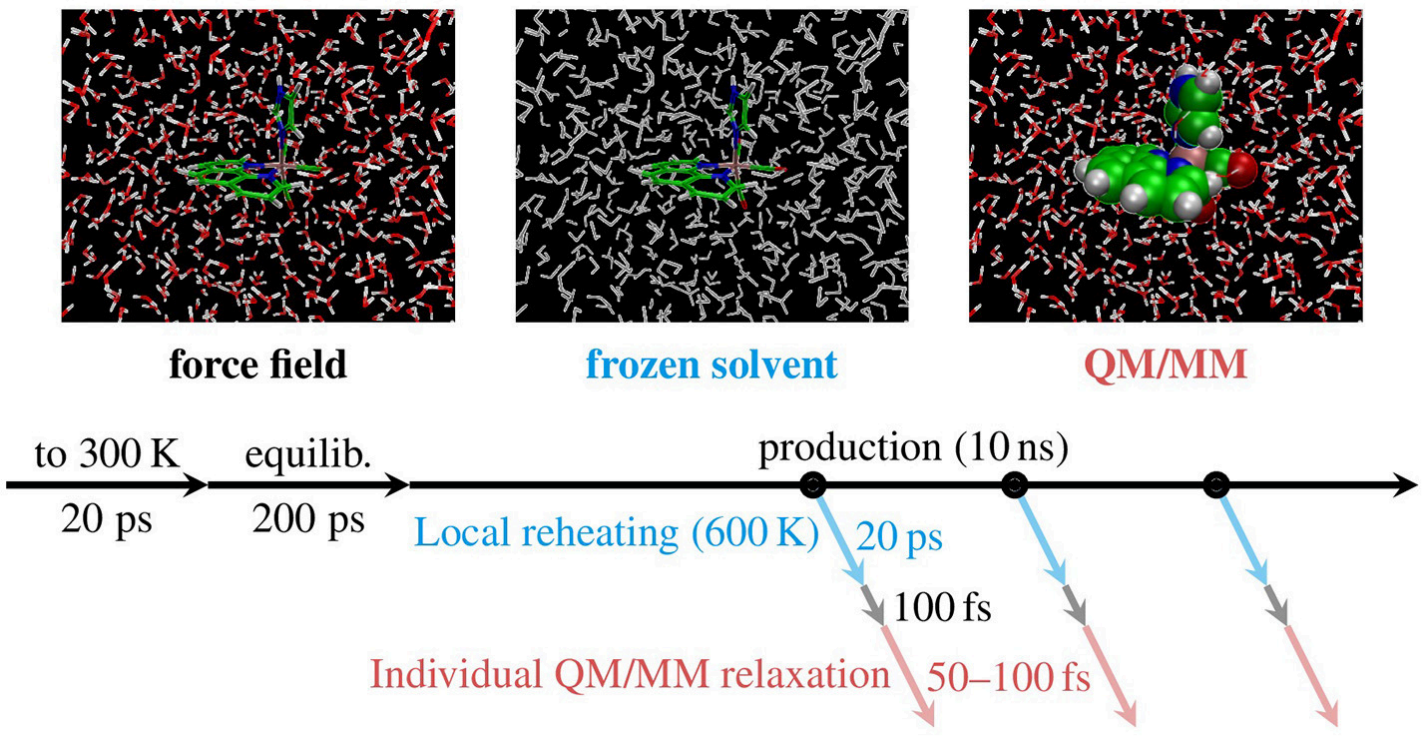
Schematic depiction of the MD sampling and adjustment procedures carried out with [Re(CO)_3_(Im)(Phen)]^+^. First, FF-based MD was carried out for thermalization (“to 300 K”), equilibration, and production. Subsequently, for each snapshot taken (black circles) we applied local temperature adjustment (blue), re-equilibration, and QM/MM-based relaxation (red), yielding 500 new snapshots.

The ground state QM/MM-MD trajectories were then propagated with SHARC, using the B3LYP/TZP+DZ(P) level of theory described above. In order to avoid any bias due to coherent motion of all trajectories after switching from GAFF to QM/MM, each of the QM/MM-MD trajectories was propagated for a randomized amount of time between 50 and 100 fs. The end points of these trajectories then represent the final initial conditions where vertical excitation calculations can be performed to compute an absorption spectrum and to select initial electronic states for nonadiabatic AIMD simulations. Figure 4 summarizes the MD-based sampling procedure and the post-processing steps described above.

## 4. Results and discussion

In this section, the effect of the procedure described above for the adjustment of the MD-sampled snapshots will be investigated. In order to quantify these effects, we will compute the distribution of several internal DOFs of [Re(CO)_3_(Im)(Phen)]^+^, as well as absorption spectra.

### 4.1. Distribution of internal coordinates

We computed the distribution of internal coordinates for the three ensembles that appeared during the adjustment steps: (i) the 500 snapshots taken directly from the 10 ns MD trajectory (denoted “FF 300 K” in the following), (ii) the 500 snapshots obtained after local temperature adjustment (“FF 600 K”), and (iii) the 500 snapshots produced at the end of the QM/MM-MD trajectories (“QM/MM”). Additionally, for comparison purposes we also calculated the distribution of internal coordinates for the “no Θ” ensemble obtained from quantum sampling (denoted “Wigner” below).

**Figure 5:**
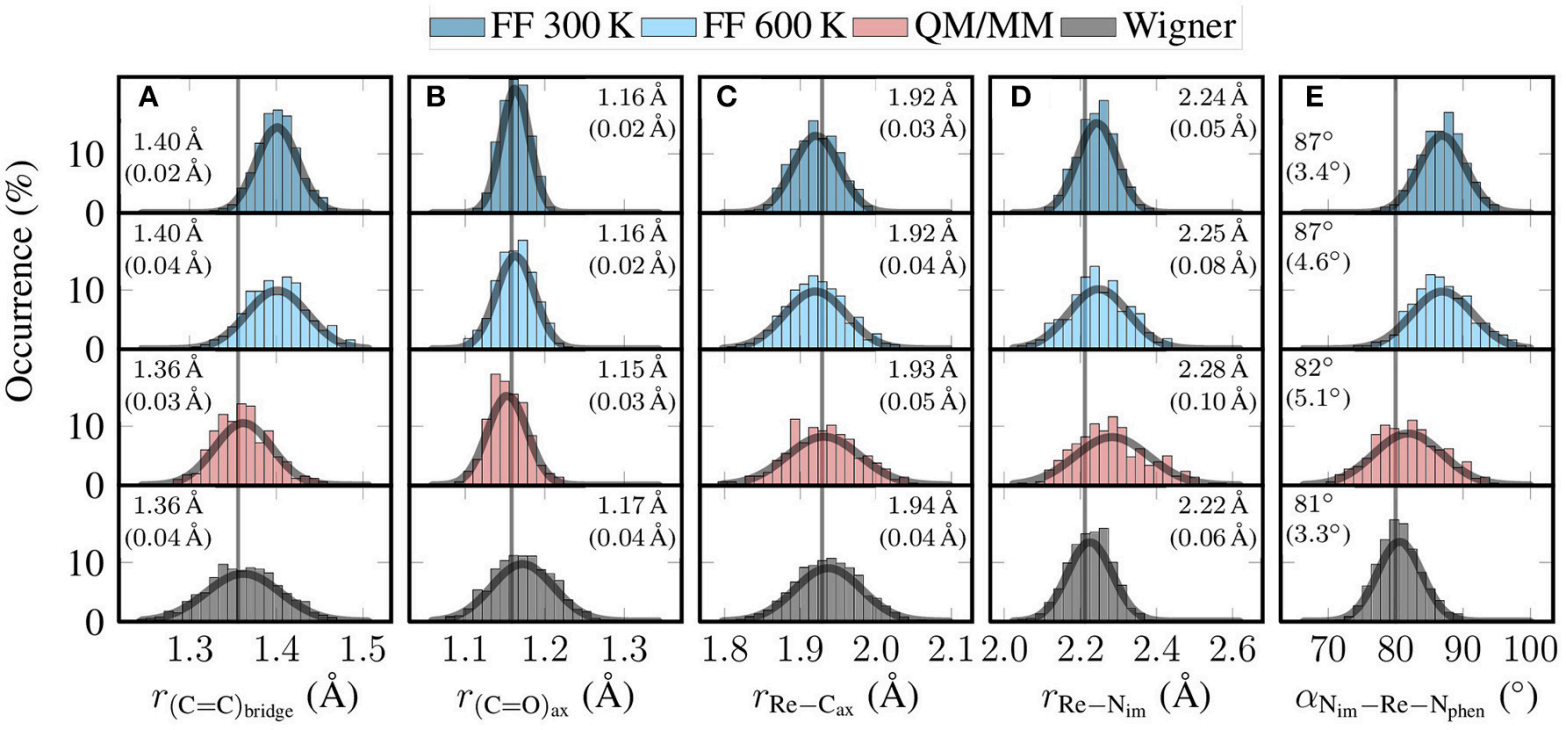
Histograms showing the distribution of key geometric parameters of [Re(CO)_3_(Im)(Phen)]^+^ for the four different ensembles (FF 300 K, FF 600 K, QM/MM, and Wigner). From left to right, we show histograms for **(A)** the length of the C = C bond of the phen bridge, **(B)** the length of the axial C = O bond, **(C)** the length of the Re–C_axial_ bond, **(D)** the length of the Re–N_im_ bond, and **(E)** the angle between the Re–N_im_ bond and the average of the Re–N_phen_ bonds. The gray curves are Gaussian fits of the distributions. The given values are the mean and (in parentheses) standard deviation of these Gaussian fits. The gray vertical lines are the optimized values (B3LYP/TZP, conformer *B*).

In Figure 5, we present histograms of the distribution of relevant geometrical parameters of [Re(CO)_3_(Im)(Phen)]^+^ as found in the four different ensembles. The figure also shows Gaussian fits, the mean and standard deviations of these Gaussians, and the optimized values. Additionally, in Figure S3 in the Supplementary Material (section 4), we show plots of the relaxation over time of the internal coordinates, and graphical depictions of the coordinates.

In Figure 5A, we plot the distribution of the length of the bond between the two bridging C atoms of the phen ligand. This C = C bond length is one example of a bond that was parametrized with GAFF. This explains the deviations between the FF-based distributions (mean of 1.40 Å) and the ab initio-based distributions (1.36 Å). It is quite satisfactory to see how the QM/MM-based relaxation step corrected this bond length to the B3LYP/TZP equilibrium value, even though only 50–100 fs of QM/MM propagation were used. As can be seen in Figure S3A, this bond performs two full oscillations in 50 fs, and is well centered on the optimized bond length between 50 and 100 fs. Figure 5A also shows that the local reheating to 600 K correctly produced a significantly broader distribution than MD at 300 K. This broader distribution is also present in the QM/MM ensemble, leading to very good overall agreement between QM/MM and Wigner distribution for this mode.

In Figure 5B we show the distributions for the axial carbonyl C = O bond length. For this bond (and for the other carbonyl bonds, too), the FF ensembles predict a mean bond length of 1.16 Å, whereas the QM/MM-MD trajectories predict a shorter value of 1.15 Å, and the Wigner distribution a value of 1.17 Å. Although the deviations are small, this disagreement of the mean values between QM/MM and Wigner is unexpected, as the C = O bonds are strong and should not exhibit significant anharmonicities. Furthermore, carbonyls in transition metal complexes are known to exhibit only weak basicity (Braga and Grepioni, 1997), so that explicit hydrogen bonds (possible in the QM/MM computations, but not in the Wigner distribution) can also be excluded as a reason for the disagreement of the mean values. Instead, a close analysis revealed in the Wigner data a slight correlation between the C = O bond length and the Re–C = O bond angle, whereas there is no such correlation for the MD-based ensembles. These correlations are presented in Figure S4 in the Supplementary Material (section 5), showing that the carbonyls become more stretched the more bent they are. This indicates that the linear normal mode assumption can lead to small biases even for modes without large-scale displacement of big atom groups.

Figure 5C shows the distribution of the length of the bond between the Re atom and the axial carbonyl C atom. This is one of the bonds that was specifically parametrized for the previously reported MD simulations (Mai et al., 2017). As can be seen, all four distributions agree quite well on the mean value, which ranges from 1.92 Å for FF 300 K to 1.94 Å for Wigner; the optimized value is 1.93 Å. This agreement, as well as Figure S3C, nicely demonstrates that the bond parametrization is accurate. The figure also shows that the width of the distribution increases from the 300 K MD run to the QM/MM-MD and Wigner ensembles, as can be expected from the increase in temperature. In particular, we want to emphasize that the widths of the QM/MM-MD and Wigner ensembles agree well with each other, even though they are based on very different assumptions (harmonic approximation on one side, MD at 600 K on the other side).

Figure 5D shows the analogous distribution for the length of the bond between Re and the imidazole N atom. Even though this bond was also parameterized from *ab initio* data, the four distributions do not agree very well with each other. The two FF ensembles show average bond lengths of 2.24–2.25 Å, whereas the QM/MM-MD trajectories produce longer bonds and the Wigner distribution shorter bonds. A possible explanation for this behavior might be that this bond is very anharmonic. This property would neither be described correctly by the Wigner distribution nor the MD runs, as the latter assume a harmonic force constant between Re and N (a minor degree of anharmonicity would be included by the FF through nonbonded interactions, though). Only the QM/MM-MD trajectory can fully describe the anharmonicity of this bond, which explains why the results of the QM/MM-MD ensemble are so different than the other ones.

In the last panel, Figure 5E, we plot the distribution of the angle between the im and phen ligands, computed as the angle between the Re–N_im_ bond and the average of the two Re–N_phen_ bonds. Its mean value is around 87° for the FF-based ensembles, but shifts to 82° for the QM/MM-MD ensemble, and to 81° for the Wigner distributions. The deviation of the FF ensembles from the QM/MM-MD and Wigner ensembles is due to the fact that this degree of freedom is coupled to the torsion mode, as conformer *B* shows an optimized value of 80° at the B3LYP/TZP level of theory, whereas conformers *A* and *A*′ have a value of 84° and 82°, respectively. This coupling is due to π stacking interactions of the two ligands, which is naturally not described by the FF. It is therefore quite satisfactory to see that the QM/MM-based relaxation trajectories are able to improve the distribution of the im–phen angle. We note, however, that according to Figure S3E, a slightly longer QM/MM relaxation time would be needed to fully relax this slow mode.

In general, the FF 300 K produces the most narrow distributions for the five shown degrees of freedom. The local temperature adjustment procedure yields significantly broadened distributions, as expected. A large effect can be seen after the QM/MM-based refinement of all individual snapshots. In this step, systematic shifts of the mean value toward the optimized values can be observed, showing that the refinement step allows correcting inaccurate FF parameters. This improvement toward the optimized values is also evidenced in Table S2 in the Supplementary Material (section 6). There, it is shown that for all CC, CN, and CO bonds, as well as all angles around Re and in the ligands, the QM/MM-based refinement significantly reduced the RMSDs between the ensemble mean and the optimized value. Only for the Re–C and Re–N bonds the QM/MM-MD ensemble does not produce distributions around the optimized values, but this is mostly due to the anharmonic Re–N_im_ bond.

As the QM/MM-MD trajectories are relatively short (50–100 fs), the refinement works best for fast degrees of freedom, as the bond lengths shown in Figures S3A–D. However, even for the low-frequency im-Re-phen angle mode in Figure S3E this short simulation time is seemingly sufficient to improve the distribution, despite the oscillation period of this mode of several hundred fs. A reason might be that such low-frequency modes experience enough friction from the solvent to damp oscillations, such that the mode converges to the equilibrium value faster than multiple oscillation periods.

### 4.2. Distribution of kinetic energy

**Figure 6:**
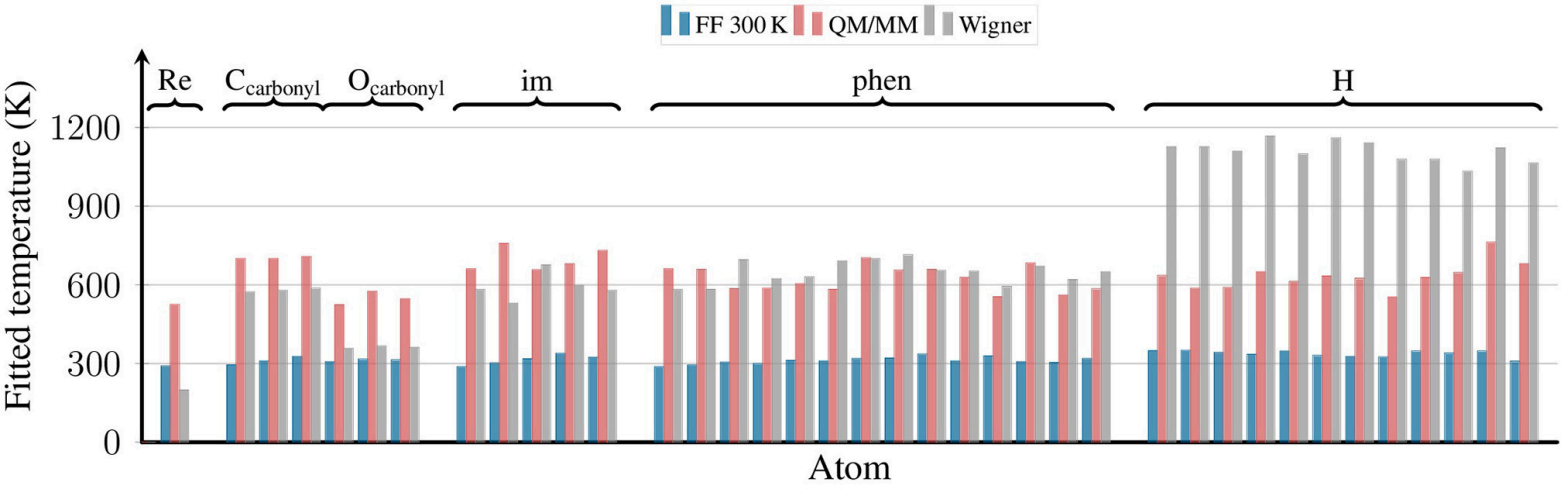
Effective temperatures for all atoms of [Re(CO)_3_(Im)(Phen)]^+^ from the FF 300 K, QM/MM, and Wigner ensembles. The effective temperatures were obtained by fitting a Maxwell-Boltzmann distribution to the distribution of kinetic energies for each atom. The plot is grouped by ligand type, with all hydrogen atoms shown on the right.

Besides scrutinizing the distribution of internal coordinates in the different ensembles, we also checked the distribution of kinetic energy among the atoms of [Re(CO)_3_(Im)(Phen)]^+^. This is shown in Figure 6, where we plot the effective temperature of each of the 38 atoms of the complex. The effective temperatures were obtained from fits of the Maxwell-Boltzmann distribution:

(3) $$\begin{array}{r} f\left(E_{\text{kin}}\right)=2\sqrt{\frac{E_{\text{kin}}}{\pi }}{\left(k_{B}T\right)}^{-\frac{3}{2}}{\text{e}}^{-\frac{E_{\text{kin}}}{k_{B}T}} \end{array}$$

to histograms of the kinetic energy distribution of the respective atoms.

The figure shows that for the FF 300 K ensemble, the kinetic energy of all atoms corresponds to 300 K, as expected for a fully equilibrated classical trajectory. The temperatures for the QM/MM-MD ensemble are clustered around 600 K, although the spread of temperatures is slightly higher than for the 300 K ensemble. This stems from the fact that the reheating step is applied to a very small system only, and temperature fluctuations in MD simulations depend on the number of atoms (Hickman and Mishin, 2016). Furthermore, these fluctuations naturally become larger for larger temperatures (Chui et al., 1992). Still, the average of the temperatures from the QM/MM-MD ensemble is very close to 600 K, showing that the re-equilibration and QM/MM-based refinement steps were chosen short enough to avoid excessive energy transfer from the reheated solute to the solvent.

The figure also shows the effective temperatures from the Wigner ensemble. As expected for quantum sampling, here the effective temperatures are not equal for all atoms. Instead, each normal mode receives energy proportional to its ZPE (plus thermal energy for low-frequency modes). We do not show the effective temperatures for the normal modes here, but also for the atoms we expect a non-equal distribution, as heavier atoms tend to contribute more to lower-frequency modes. This non-equal distribution can nicely be seen in Figure 6: the Re atom has a very low effective temperature of about 200 K, the C and N atoms of phen and im of about 600 K, and the H atoms of about 1,100 K. Interestingly, while the carbonyl C atoms also have effective temperatures of about 600 K, the oxygen atoms have a much lower value of 360 K.

In general, it is very reassuring to observe that the effective temperature of the second row atoms (C, N, O) is well reproduced by the locally reheated MD simulation (QM/MM-MD ensemble). This is particularly important because the motion of the second row atoms is to a large portion determining the excited-state dynamics of complexes like [Re(CO)_3_(Im)(Phen)]^+^, for example through the influence of bond length alterations on the im and phen π systems. On the contrary, the QM/MM-MD ensemble does not correctly predict the kinetic energies of the H atoms. However, this problem is mostly related to the X–H stretch motions, and it is likely that these stretch motions are less relevant for the electronically excited-state evolution. Hence, it seems that in the particular case investigated here—[Re(CO)_3_(Im)(Phen)]^+^ in water—locally heating the chromophore to 600 K provides kinetic energies that are as close to the ZPE as possible for a classical ensemble.

### 4.3. Broadened absorption spectra

Based on the three MD-based ensembles that were presented above—FF 300 K, FF 600 K, and QM/MM—we computed absorption spectra using B3LYP/DZ(P) and electrostatic embedding; 30 singlet states (see section Computational Details) were computed for each snapshot. No spectrum is shown for the Wigner sampling, since it cannot produce coordinates for the MM point charges which are required for electrostatic embedding. We also do not show a spectrum for Wigner sampling and implicit solvation, because it has been previously shown (Mai et al., 2017) that changing the embedding technique has a large effect on the spectrum, and that effect would conceal the effect of the sampling.

**Figure 7:**
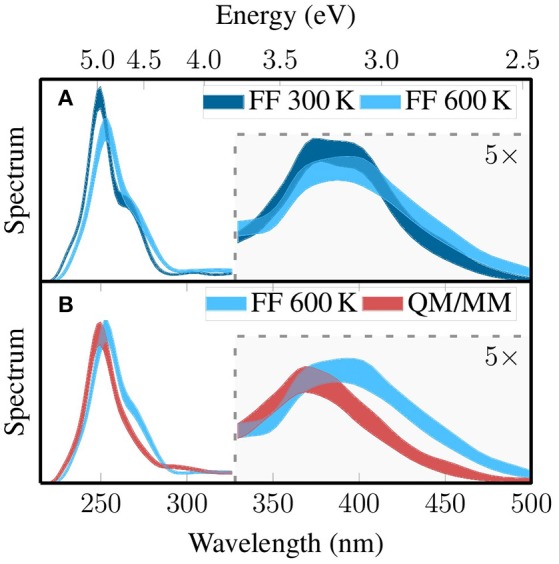
Pairwise comparison of the simulated absorption spectra based on the three MD-based ensembles discussed above: FF 300K with FF 600K **(A)** and FF 600K with QM/MM **(B)**. The spectrum of the Wigner ensemble is not shown because it does not contain MM point charges, which would be required to calculate comparable spectra. The thickness of the line quantifies the error due to finite sampling size, such that the spectrum from complete sampling lies within the thick line with 99.7% probability (3σ). The part of the figure that is highlighted with gray shading has been multiplied by a factor of five.

The three spectra are shown in Figure 7, where in Figure 7A we compare the spectrum before and after local temperature adjustment (FF 300 K and FF 600 K) and in Figure 7B we compare the spectra before and after QM/MM-based refinement. In order to also consider the random errors in the spectra originating from the finite size of our ensembles (500 geometries each), we also performed bootstrapping for each of the three spectra. During bootstrapping for one spectrum, we drew 500 geometries randomly (with replacement) from the 500 geometries in the ensemble, and based on the 500 drawn geometries (one bootstrap resample) we computed an absorption spectrum. We then repeated this process 100 times, giving 100 random absorption spectra, for which we computed for each wave length the standard deviation. In the figure, we plot colored areas that are delimited by mean plus/minus three standard deviations (3σ) of the bootstrapped spectra, which means that the spectrum corresponding to complete sampling has a 99.7% probability of lying within the colored area. Generally, we note that the sampling errors are nearly constant and small over the whole range, showing that for the [Re(CO)_3_(Im)(Phen)]^+^ system, 500 snapshots seems to be enough to sample the absorption spectra adequately.

Each of the absorption spectra consists basically of two absorption bands (Mai et al., 2017). The first band is located between 320 and 500 nm (2.5–3.8 eV) and is due to a few low-lying metal-to-ligand charge transfer (MLCT) states. The second band is between 230 and 300 nm (4.1–5.4 eV) and arises from absorption of several very bright intra-ligand (IL) transitions localized on the phen. All three spectra reproduce these general features.

While the spectra are generally similar, clear effects of the sampling strategy on the position and width of the absorption bands can be observed. The most important effects of the local temperature adjustment (Figure 7A) are a broadening of the MLCT band with increased absorption around 2.75 eV (450 nm), a shift of the IL absorption maximum from 5.0 to 4.9 eV accompanied by reduction in intensity, and a weakening of the shoulder around 4.6 eV. These effects are all consistent with the expected broadening of the whole spectrum due to the increased temperature. The QM/MM-based refinement step leads to a significant blue shift of the MLCT band from around 3.2–3.4 eV and a slight reduction in intensity. Furthermore, the IL band is shifted from 4.9 to 5.0 eV These effects are mostly due to the adjustment of the bond lengths toward the B3LYP equilibrium values. In total, Figure 7 clearly shows that our sampling protocols have a large effect on the computed spectra. The shift of the MLCT band by relaxing the bond lengths—0.2 eV—is comparable to typical errors of widely employed electronic structure methods, for example the mean absolute deviation of 0.27 eV of TD-B3LYP reported by Silva-Junior et al. (2008). This shows unambiguously that proper configurational sampling in indispensable for accurate spectrum simulations.

A slightly different perspective on the effect of the sampling on the excited states is given by the density-of-state spectra that are reported in Figure S5 in the Supplementary Material (section 7). There, it is shown that the local temperature adjustment only leads to minor red-shifting of all states. However, the individual QM/MM-based refinement leads to a significant blue shift of the density of states. This has the consequence that in the spectroscopically important region of 400 nm and longer the density of states is reduced, which might have a large effect on the excited-state dynamics occurring after excitation.

## 5. Conclusion

We have presented computational results on the phase space sampling of [Re(CO)_3_(Im)(Phen)]^+^ (Im, imidazole; Phen, phenanthroline), which is a metal complex featuring an almost barrierless torsion of the monodentate Im ligand. These results show that the phase space (Wigner) distribution of this molecule cannot be correctly sampled using a linear normal mode harmonic oscillator—a popular approach that is often used for sampling such distributions for small molecules (Dahl and Springborg, 1988; Schinke, 1995; Barbatti and Sen, 2016). An alternative way to sample the phase space of this complex is thus based on classical molecular dynamics (MD). Unfortunately, classical sampling also suffers from limitations, most importantly (i) the inability to properly describe zero-point energy (ZPE) which leads to too narrow distributions and too little kinetic energy, and (ii) the inaccuracy of the generally employed classical force fields.

Here, we have reported two new protocols intended to mitigate these two limitations. A prerequisite for both approaches is a completed MD trajectory of sufficient length (tenth to hundreds of nanoseconds) from which a number of snapshots is taken; both protocols are then applied to these snapshots as post-processing steps. The first protocol, called “local temperature adjustment,” can be summarized as running MD simulations starting from the snapshots, with the environment frozen and the thermostat set to a temperature corresponding to the ZPE of the molecule. In this way, the internal energy of the chromophore can be made closer to the ZPE, while avoiding overheating of the—possibly biological—environment. This is especially relevant if the phase space sampling is intended to provide initial conditions for excited-state dynamics simulations, because these simulations might produce wrong results if the internal energy of the chromophore is too low (e.g., too slow dynamics, missing reaction paths).

The second protocol, termed “individual QM/MM-based refinement,” basically consists of running a short QM/MM-MD (or another *ab initio* method for large systems) trajectory with each of the snapshots as starting point. These short trajectories allow the system to relax from the possibly inaccurate force field equilibrium bond lengths/angles to the desired ab initio values. Naturally, the longer these QM/MM-MD trajectories are run, the more complete the relaxation. Generally, stiff bond lengths and angles will relax within a few hundred femtoseconds or less, but will already oscillate around the correct values within tens of femtoseconds. Hence, a practical detail for the “individual QM/MM-based refinement” approach is to run the QM/MM-MD trajectories for a randomized amount of time, e.g., between 50 and 100 fs, in order to avoid a sampling bias due to coherent motion induced by the switch from force field to *ab initio* potential. The slow degrees of freedom—like solvation shells or low-frequency modes of flexible molecules—will then be described at the force field level.

The “individual QM/MM-based refinement” should be superior compared to the usual approach of running one few-picoseconds QM/MM-MD trajectory and taking snapshots only from this relatively short time window (Domingo et al., 2012; Valsson et al., 2013). The individual approach on one side provides a much less biased sampling of the environment, as the snapshots can be taken from a many-nanoseconds MD trajectory instead of a few-picoseconds trajectory where all snapshots will be correlated. On the other side, the individual approach is computationally not more expensive than the long QM/MM-MD trajectory and can even be more easily parallelized.

Finally, here we have presented results of the application of the two new protocols to the sampling of [Re(CO)_3_(Im)(Phen)]^+^ in water. The results show that bond lengths and angles are distributed well around the *ab initio* equilibrium values, and the kinetic energies of many atoms are distributed similarly to a Wigner distribution. We have also shown that these two sampling strategies can have a significant effect on absorption spectrum and density of states, for example introducing shifts of absorption bands by up to 0.2 eV, as well as on the results of excited-state dynamics simulations.

## Author contributions

SM, AM, and LG conceived this research project. HG and SM performed the classical MD simulations, and all other simulations were performed by SM. AM and SM created a first draft of the manuscript, and all authors contributed to the writing of the final manuscript.

### Conflict of interest statement

The authors declare that the research was conducted in the absence of any commercial or financial relationships that could be construed as a potential conflict of interest.

## Supplementary material

Optimized coordinates, verification of numerical integration quality, definition of the torsional angle Θ of [Re(CO)_3_(Im)(Phen)]^+^, plots of internal coordinate relaxation, carbonyl bond length plots, table with RMSD values from the different ensembles, and density-of-state spectra.
