# Supplementary material for: Novel Molecular-Dynamics-Based Protocols for Phase Space Sampling in Complex Systems
Source: Front Chem. 2018 Oct 17;6:495. doi: 10.3389/fchem.2018.00495 (PMC6199692; doi:10.3389/fchem.2018.00495)
Supplement: Supplementary file 1 [file Data_Sheet_1.PDF]

Supplementary Material: A novel molecular-dynamics-based protocol for phase space sampling in complex systems

1 OPTIMIZED GEOMETRIES

These geometries were obtained with B3LYP/TZP and COSMO(water). The frequency calculations for constructing the Wigner ensemble are based on these geometries.

```
38
Conformer A (-11.282796 Hartree)
Re -0.732324 -1.918468 +0.000000
N +1.474048 -1.672488 +0.000000
N -0.742092 -0.166646 +1.345378
N -0.742092 -0.166646 -1.345378
N +3.493185 -0.827385 +0.000000
C +2.179664 -0.547863 +0.000000
C -2.659628 -2.038943 +0.000000
C -0.635115 -3.275924 -1.367477
C -0.635115 -3.275924 +1.367477
C -0.712896 -0.186340 +2.676171
C -0.734789 +0.981737 +3.446552
C -0.787285 +2.206021 +2.822312
C -0.813388 +2.263346 +1.415759
C -0.790158 +1.042257 +0.714246
C -0.790158 +1.042257 -0.714246
C -0.813388 +2.263346 -1.415759
C -0.787285 +2.206021 -2.822312
C -0.734789 +0.981737 -3.446552
C -0.712896 -0.186340 -2.676171
C -0.851866 +3.490208 +0.678898
C -0.851866 +3.490208 -0.678898
C +3.651774 -2.192915 +0.000000
C +2.392507 -2.712062 +0.000000
O -3.816023 -2.106979 +0.000000
O -0.568084 -4.071195 -2.207966
O -0.568084 -4.071195 +2.207966
H -0.672613 -1.157258 +3.146344
H -0.708485 +0.899689 +4.523484
H -0.708485 +0.899689 -4.523484
H -0.672613 -1.157258 -3.146344
H -0.874745 +4.420485 +1.231425
H -0.874745 +4.420485 -1.231425
H -0.804257 +3.123461 +3.395982
H -0.804257 +3.123461 -3.395982
H +4.233274 -0.141870 +0.000000
H +2.080025 -3.740651 +0.000000
H +1.786889 +0.451561 +0.000000
H +4.619635 -2.660225 +0.000000
```

```
38
Conformer B (-11.283470 Hartree)
Re -0.506395 +0.945575 +0.234079
N +1.079487 +0.145454 +1.563875
N +0.827189 +0.166915 -1.343102
N -1.004054 -1.199681 +0.089195
N +3.043752 -0.394787 +2.360920
C +2.389046 +0.316956 +1.429238
C -1.870529 +1.487599 -1.021502
C -1.673980 +1.368359 +1.711172
C +0.202023 +2.739280 +0.279315
C +1.705997 +0.861606 -2.063615
C +2.573899 +0.257142 -2.978953
C +2.531295 -1.106846 -3.153120
C +1.607482 -1.869358 -2.414371
C +0.765517 -1.183940 -1.516808
C -0.201690 -1.908372 -0.754702
C -0.302881 -3.305979 -0.897483
C -1.275870 -3.971016 -0.128168
C -2.085801 -3.243067 +0.712356
C -1.922165 -1.856492 +0.795174
C +1.489098 -3.291121 -2.535091
C +0.574689 -3.980307 -1.806056
C +2.121697 -1.057789 +3.135934
C +0.901464 -0.716221 +2.634855
O -2.687066 +1.801289 -1.780771
O -2.380718 +1.594744 +2.601578
O +0.657261 +3.805221 +0.293662
H +1.725007 +1.929819 -1.906350
H +3.267490 +0.874563 -3.531093
H -2.845065 -3.718973 +1.315855
H -2.544397 -1.265105 +1.450402
H +2.147085 -3.804816 -3.223860
H +0.488997 -5.054904 -1.900940
H +3.196185 -1.600147 -3.850026
H -1.377920 -5.045398 -0.207016
H +4.046436 -0.432366 +2.465816
H -0.075649 -1.025674 +2.958572
H +2.873511 +0.926350 +0.688387
H +2.407902 -1.696681 +3.951531
```

```
38
Conformer A' (-11.282805 Hartree)
Re -0.751127 -1.918225 +0.000000
N +1.456751 -1.655025 +0.000000
N -0.751764 -0.167608 +1.345532
N -0.751764 -0.167608 -1.345532
N +3.587033 -2.161731 +0.000000
C +2.336944 -2.650402 +0.000000
C -2.678090 -2.035687 +0.000000
C -0.657231 -3.275352 -1.367570
C -0.657231 -3.275352 +1.367570
C -0.717737 -0.188228 +2.676094
C -0.715276 +0.979907 +3.446539
C -0.747026 +2.204889 +2.822216
C -0.778558 +2.262620 +1.415799
C -0.782276 +1.041454 +0.714348
C -0.782276 +1.041454 -0.714348
C -0.778558 +2.262620 -1.415799
C -0.747026 +2.204889 -2.822216
C -0.715276 +0.979907 -3.446539
C -0.717737 -0.188228 -2.676094
C -0.790824 +3.490093 +0.678869
C -0.790824 +3.490093 -0.678869
C +3.521221 -0.788992 +0.000000
C +2.194271 -0.480933 +0.000000
O -3.834718 -2.101797 +0.000000
O -0.590993 -4.070711 -2.208207
O -0.590993 -4.070711 +2.208207
H -0.691665 -1.159807 +3.145940
H -0.684768 +0.897352 +4.523329
H -0.684768 +0.897352 -4.523329
H -0.691665 -1.159807 -3.145940
H -0.791368 +4.420596 +1.231408
H -0.791368 +4.420596 -1.231408
H -0.741826 +3.122484 +3.395818
H -0.741826 +3.122484 -3.395818
H +4.428874 -2.717782 +0.000000
H +1.735533 +0.489466 +0.000000
H +2.100193 -3.698715 +0.000000
H +4.399342 -0.168991 +0.000000
```

2 DFT NUMERICAL INTEGRATION QUALITY

For all DFT and TD-DFT calculations described in the main manuscript, we employed specifically adjusted settings for the numerical integration quality in ADF. In particular, for the Becke numerical integration grid (Franchini et al., 2013), heavy atoms were treated with **good** quality, whereas H atoms used **normal** quality. For the ZlmFit Coulomb fit method (Franchini et al., 2014), the metal atom, the first coordination sphere, and the oxygen atoms used **good** quality, other heavy atoms used **normal** quality, and hydrogens used **basic** quality. Finally, for the RI Hartree-Fock scheme (Krykunov et al., 2009)—which is the main bottleneck in ADF calculations with hybrid functionals—**basic** quality was used for heavy atoms and "**verybasic**" quality for H atoms.

In order to judge the effect of these quality settings on the electronic structure calculations, we performed single point plus gradient calculations for one randomly chosen snapshot from the $[Re(CO)_3(Im)(Phen)]^+$ ensemble. Using this geometry (including the point charges), we performed a calculation using the above-mentioned settings and a reference calculation employing **good** quality for Becke grid and ZlmFit and **normal** quality for RI-HF. The settings are summarized in the first section of Table S1.

The computations included 6 excited singlet and 8 triplet states. In order to estimate the effort for excited-state gradient calculations (relevant for dynamics simulations), we also computed the gradients of S_3, S_4, S_5, T_3, T_4, and T_5; this resembles a typical time step request in a SHARC trajectory.

The obtained timings (obtained on 10 cores of an Intel® Xeon E5-2650 v3) for the two calculations are shown in Table S1. It can be seen that the reduced settings lead to a significant decrease in computational effort, cutting the wallclock time roughly in half. In particular, the Davidson step profits massively from the reduced settings. We note here that since the execution of these benchmark computations, the ADF code was improved to further reduce the timings for multiple gradient computations.

Table S1 also shows that the reduced settings have only a small influence on the vertical excitation energies of the computed states. Compared to the reference data, we obtained a bias of all energies of -0.03 eV, combined with a random error of 0.01 eV. These encouraging observations are further validated by the small differences in the oscillator strengths of the singlet states. Especially the latter show that the reduced settings do not lead to state reordering or significant changes in the excited-state characters.

Finally, Table S1 shows the effect of the quality reduction on the gradients, which are usually more sensitive to the quality of integration. As can be seen, the errors in the gradients amount to only a few percent of the gradients. The good agreement is more easily visible in Figure S1, which compares the vector elements for the QM region.

In summary, these data show that the reduced numerical accuracy settings in our calculations do not lead to notable errors in the results, but allow cutting all computation times in half.

Table S1. Comparison of the excitation calculation between the reference data and the employed settings.

	Reference	Employed settings
— Settings —		
Becke grid (Re, C, N, O)	good	good
Becke grid (H)	good	normal
ZlmFit (Re, $(CO)_3$, N_{coord})	good	good
ZlmFit (other C, N)	good	normal
ZlmFit (H)	good	basic
RI-HF (Re, C, N, O)	normal	basic
RI-HF (H)	normal	verybasic
Davidson convergence (E_h)	10^{-7}	10^{-6}
— Timings —		
Time for SCF (s)	360	180
Time for Davidson (s)	2621	529
Time for Gradients (s)	5601	3281
Time for all (s)	9398	4433
Speedup (%)	100	212
— Vertical excitation energies (eV) —		
S_1	2.739	2.702
S_2	2.932	2.894
S_3	3.046	3.007
S_4	3.180	3.141
S_5	3.271	3.237
S_6	3.394	3.355
T_1	2.526	2.514
T_2	2.617	2.594
T_3	2.719	2.692
T_4	2.916	2.879
T_5	3.007	2.978
T_6	3.164	3.128
T_7	3.276	3.253
T_8	3.397	3.399
Bias	0	0.029
Standard deviation	0	0.011
— Oscillator strength —		
S_1	0.0012	0.0012
S_2	0.0043	0.0039
S_3	0.0280	0.0268
S_4	0.0160	0.0157
S_5	0.1063	0.1057
S_6	0.0189	0.0205
— Gradients —		
RMSD Grad. S_3 (a.u.)	0	0.0006
RMSD Grad. S_4 (a.u.)	0	0.0006
RMSD Grad. S_5 (a.u.)	0	0.0007
RMSD Grad. T_3 (a.u.)	0	0.0010
RMSD Grad. T_4 (a.u.)	0	0.0006
RMSD Grad. T_5 (a.u.)	0	0.0007
Relative error Grad. S_3 (%)	0	1.9
Relative error Grad. S_4 (%)	0	1.9
Relative error Grad. S_5 (%)	0	2.2
Relative error Grad. T_3 (%)	0	3.0
Relative error Grad. T_4 (%)	0	1.9
Relative error Grad. T_5 (%)	0	2.3

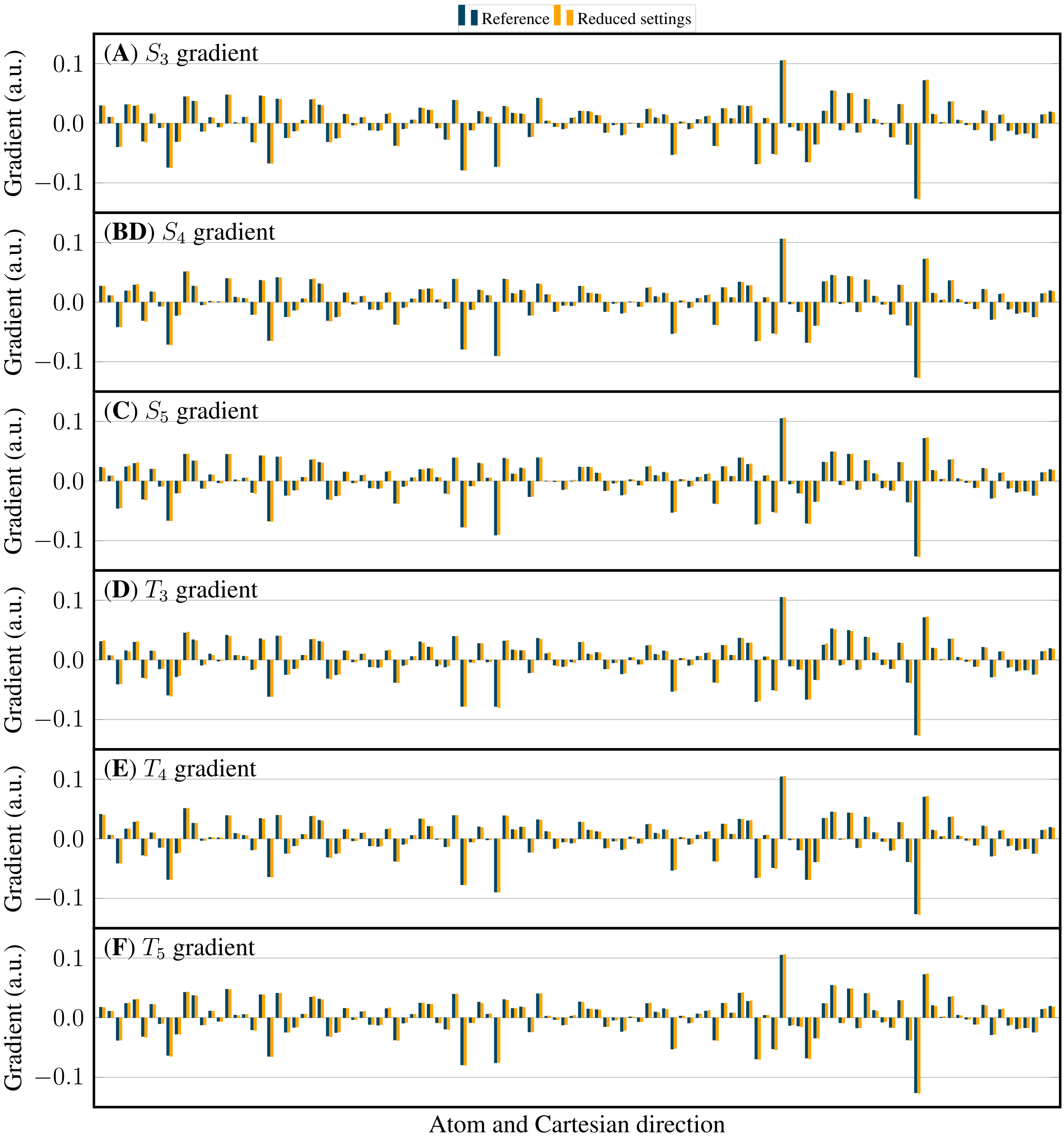

Figure S1. Comparison of the gradients of S_3, S_4, S_5, T_3, T_4, and T_5. Note that these gradients also include the electrostatic and Van-der-Waals contributions.

3 DEFINITION OF TORSION ANGLE Θ

We use the following definition of the torsion angle for the imidazole (Im) ligand of $[Re(CO)_3(Im)(Phen)]^+$ (see Figure S2 for definition of the dihedrals Φ_1 and Φ_2):

$$\Theta = \frac{\Phi_1 + \Phi_2}{2} + \begin{cases} -90 & \text{, if } \Phi_1 < \Phi_2 \\ 90 & \text{, else} \end{cases} \tag{S1}$$

If the torsion angle Θ is not in the interval from $-180°$ to $180°$, it is shifted by $\pm 360°$ accordingly. With this definition, conformer A is at $-90°$, A′ is at $+90°$, and conformer B is at approximately $0°$ and $\pm 180°$.

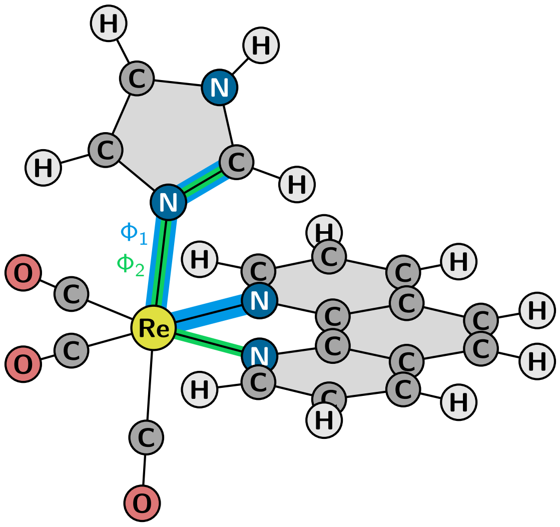

Figure S2. Definition of the dihedral angles Φ_1 and Φ_2. The first atom in the dihedral definition is in both cases the carbon atom at the Im ligand. See equation (S1) for a definition of the torsion angle Θ.

4 TIME SCALE OF RELAXATION DURING QM/MM DYNAMICS

In Figure S3, we show plots of the evolution of the internal coordinates that are plotted in Figure 5 in the main manuscript. The figure shows that the simulated time (50–100 fs) is sufficient to relax all bond lengths, from the fast oscillating C=O bonds to the much slower Re–N bonds, whereas the large-scale relative motion of im and phen is partially relaxed.

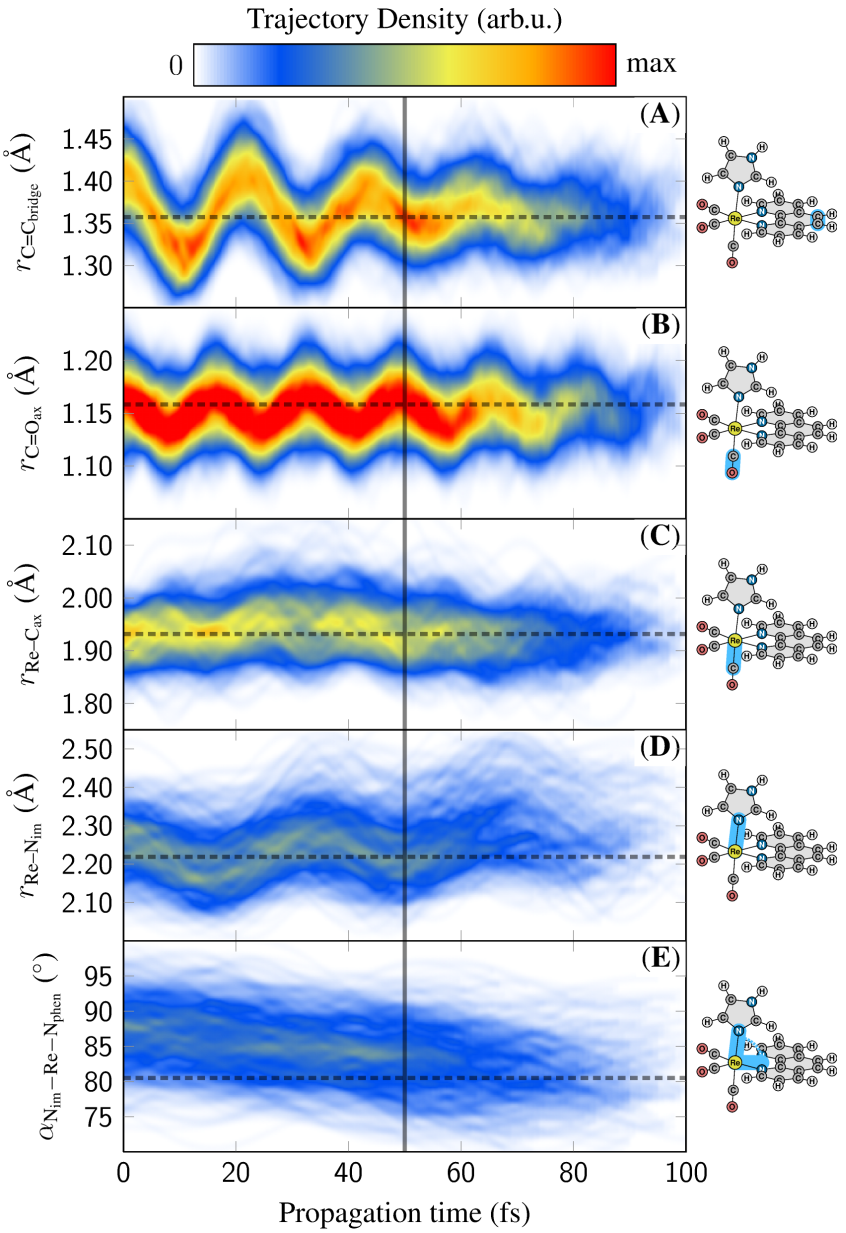

Figure S3. Depiction of the temporal evolution of the five internal degrees of freedom that are discussed in the main text (see Figure 5). The vertical black line denotes the 50–100 fs interval from which the snapshots were taken, and the dashed horizontal line denotes the optimized value (conformer B, B3LYP/TZP+DZ(P)). For each internal coordinate (**A** to **E**), the plot shows the density of trajectories, computed by Gaussian convolution of all bond parameters of all 500 QM/MM trajectories. On the right, the plotted internal coordinate is depicted: (**A**) C=C bond length between the outer "bridge" C atoms of phen, (**B**) bond length of the axial C=O, (**C**) axial Re–C bond length, (**D**) Re–N bond length to the im ligand, and (**E**) angle between the Re–N_{im} bond and the average of the two Re–N_{phen} bonds.

5 BOND LENGTH OF CARBONYLS

In Figure S4, we show that for the QM/MM ensemble the bond length of the carbonyl ligands is completely uncorrelated from the Re–C=O bond angle. On the contrary, for the Wigner ensemble, there is a slight correlation, such that for smaller angles the bond becomes longer. This is due to the assumption of linear normal modes in the Wigner sampling. On the right of the figure, we show a sketch and expression giving the theoretical relation between angle and bond length, under the assumption that C and O are displaced perpendicularly to the center line by equal amounts x.

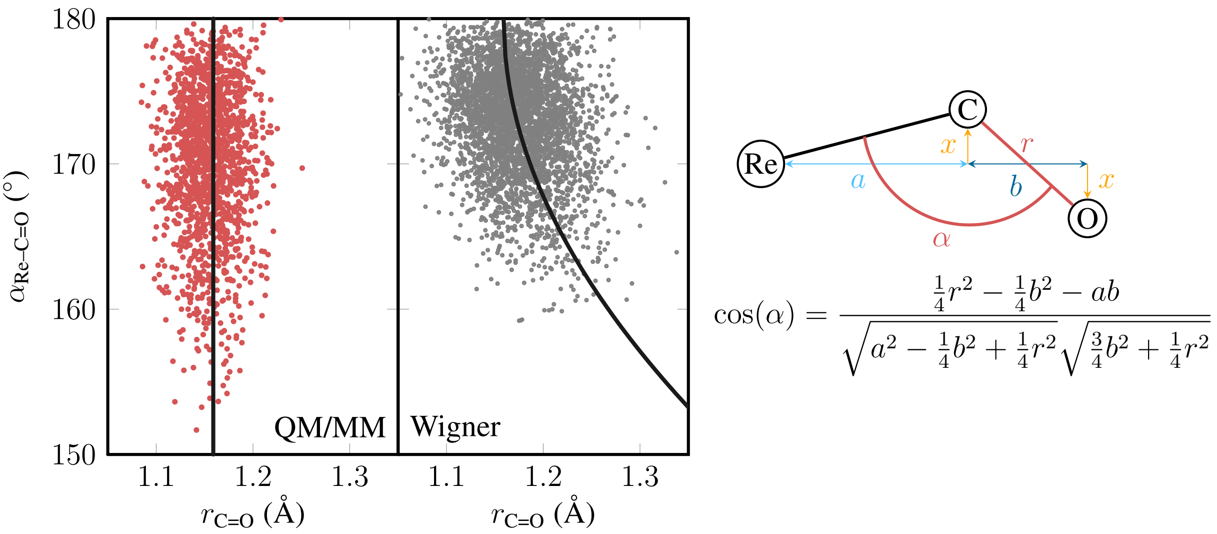

Figure S4. Scatter plots correlating the carbonyl bond lengths with the Re–C=O angles (all three carbonyls are plotted). The plot on the left is for the QM/MM ensemble, on the right for the Wigner ensemble. The black line on the left denotes the equilibrium value. The black line on the right follows the expression for $\cos(\alpha)$, which is given on the far right, for $a = 1.937$Å and $b = 1.159$Å.

6 RMSD OF BOND LENGTHS AND ANGLES

Besides the five internal coordinates appearing in Figure 5 of the main manuscript, we also scrutinized the distributions of all other bond length and angle values from the different ensembles. In particular, we investigated the following categories of bond parameters: (i) the six bond lengths involving Re, (ii) the 24 bond lengths between C, N, and O atoms, (iii) the 12 bond lengths involving H atoms, (iv) the 12 cis bond angles with Re as central atom, and (v) the 23 angles involving the C and N atoms of phen or im. In Table S2 we compile the RMSDs from the optimized values for these five categories including 77 internal coordinates.

Table S2. RMSDs between ensemble means and optimized values for 5 categories of internal coordinates of $[Re(CO)_3(Im)(Phen)]^+$. Bond length RMSDs are given in Å, angle RMSDs in degree.

Type of coordinate	#coordinates	— RMSD from optimized —			
		FF 300K	FF 600K	QM/MM	Wigner
ReC and ReN bonds	6	0.020	0.022	0.038	0.005
CC, CN, CO bonds	24	0.021	0.022	0.010	0.009
CH and NH bonds	12	0.010	0.012	0.014	0.021
Angles around Re	12	2.73	2.83	0.86	0.30
Angles in phen and im	23	1.71	1.82	0.40	0.20

The table shows the differences between the four ensembles discussed in the main text. It can be seen that for the bond lengths involving Re, the force field was parametrized reasonably well, with a difference of 0.02 Å. For these bonds, the QM/MM calculations predict on average longer Re–N bonds than the optimized values, which is due to the anharmonicity of these bonds. For the CC, CN, and CO bonds, the QM/MM refinement improves the bond lengths significantly, compared to the force field. Also for the bond angles of the Re coordination sphere and of the ligands, the QM/MM refinement leads to much better agreement with the optimized values.

7 DENSITY OF STATE SPECTRA

In Figure S5 we show the density-of-state spectra of $[Re(CO)_3(Im)(Phen)]^+$ based on the three MD-based ensembles. In panel (**A**), we compare the FF 300K and FF 600K spectra. It can be seen that they are in principle quite similar. However, the spectrum after reheating (FF 600K) is shifted to lower energies—it extends to lower energies, while the high-energy band is red-shifted. Furthermore, the FF 600K spectrum shows less structure, even though both spectra were convoluted with Gaussians of the same width.

In panel (**B**), we compare the density-of-state spectra after reheating with the one after QM/MM refinement. The relaxation of the bond lengths and angles by the refinement lead to a significant change in the density of states of the complex. In particular, the low-energy states were strongly shifted to higher energies.

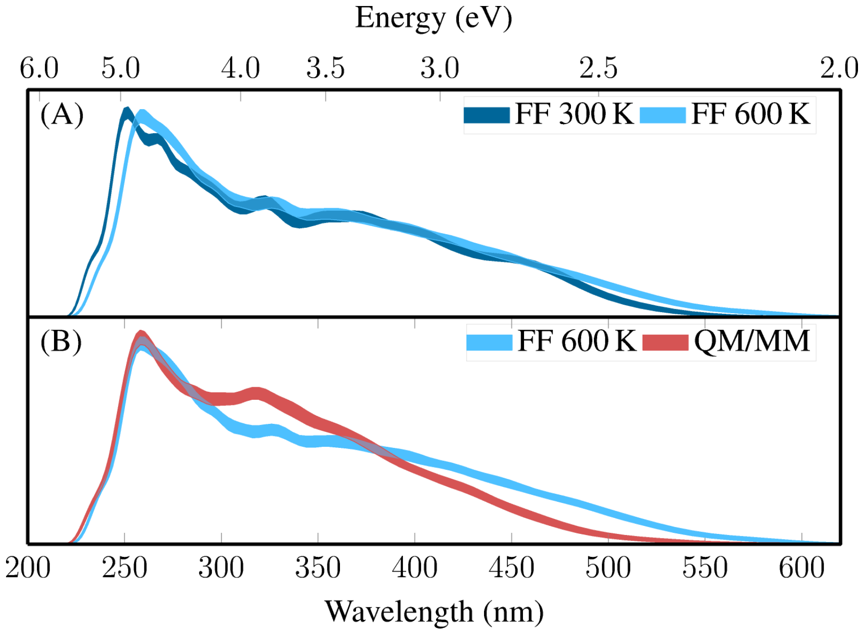

Figure S5. Pairwise comparison of the simulated density-of-state spectra based on the three MD-based ensembles discussed above: FF 300 K, FF 600 K, and QM/MM. The thickness of the line quantifies the error due to finite sampling size, such that the spectrum from complete sampling lies within the thick line with 99.7% probability (3σ). The density of states considers 120 excited states (30 singlets and 3×30 triplet states).

SUPPLEMENTARY REFERENCES

Franchini, M., Philipsen, P. H. T., van Lenthe, E., and Visscher, L. (2014). Accurate coulomb potentials for periodic and molecular systems through density fitting. *J. Chem. Theory Comput.* 10, 1994–2004. doi:10.1021/ct500172n

Franchini, M., Philipsen, P. H. T., and Visscher, L. (2013). The Becke fuzzy cells integration scheme in the Amsterdam Density Functional program suite. *J. Comput. Chem.* 34, 1819–1827. doi:10.1002/jcc.23323

Krykunov, M., Ziegler, T., and van Lenthe, E. (2009). Hybrid density functional calculations of nuclear magnetic shieldings using Slater-type orbitals and the zeroth-order regular approximation. *Int. J. Quantum Chem.* 109, 1676–1683. doi:10.1002/qua.21985
